# Comparison of treatments for the prevention of fetal growth restriction in obstetric antiphospholipid syndrome: a systematic review and network meta-analysis

**DOI:** 10.1007/s11739-020-02609-4

**Published:** 2021-01-21

**Authors:** Maria Letizia Urban, Alessandra Bettiol, Irene Mattioli, Giacomo Emmi, Gerardo Di Scala, Laura Avagliano, Niccolò Lombardi, Giada Crescioli, Gianni Virgili, Caterina Serena, Federico Mecacci, Claudia Ravaldi, Alfredo Vannacci, Elena Silvestri, Domenico Prisco

**Affiliations:** 1 Department of Experimental and Clinical Medicine, University of Florence, Florence, Italy; 2 Department of Health Sciences, San Paolo Hospital Medical School, University of Milan, Milan, Italy; 3 Department of Neurosciences, Psychology, Drug Research and Child Health, University of Florence, Florence, Italy; 4 PeaRL Perinatal Research Laboratory, University of Florence, CiaoLapo Foundation for Perinatal Health, Prato, Italy; 5 Division of Obstetrics and Gynaecology, Department of Biomedical, Experimental and Clinical Sciences, University of Florence, Florence, Italy; 6 Department of Health Sciences, University of Florence, Florence, Italy; 7 Centre for Public Health, Queen’s University Belfast, Belfast, Northern Ireland

**Keywords:** Antiphospholipid syndrome, Fetal growth restriction, Aspirin, Heparin, Network meta-analysis

## Abstract

Women with criteria and non-criteria obstetric antiphospholipid syndrome (APS) carry an increased risk of pregnancy complications, including fetal growth restriction (FGR). The management of obstetric APS traditionally involves clinicians, obstetricians and gynaecologists; however, the most appropriate prophylactic treatment strategy for FGR prevention in APS is still debated. We performed a systematic review and network meta-analysis (NetMA) to summarize current evidence on pharmacological treatments for the prevention of FGR in APS. We searched PubMed and Embase from inception until July 2020, for randomized controlled trials and prospective studies on pregnant women with criteria or non-criteria obstetric APS. NetMA using a frequentist framework were conducted for the primary outcome (FGR) and for secondary outcomes (fetal or neonatal death and preterm birth). Adverse events were narratively summarised. Out of 1124 citations, we included eight studies on 395 pregnant patients with obstetric APS treated with low-dose aspirin (LDA) + unfractionated heparin (UFH) (*n* = 132 patients), LDA (*n* = 115), LDA + low molecular weight heparin (*n* = 100), LDA + corticosteroids (*n* = 29), LDA + UFH + intravenous immunoglobulin (*n* = 7), or untreated (*n* = 12). No difference among treatments emerged in terms of FGR prevention, but estimates were largely imprecise, and most studies were at high/unclear risk of bias. An increased risk of fetal or neonatal death was found for LDA monotherapy as compared to LDA + heparin, and for no treatment as compared to LDA + corticosteroids. The risk of preterm birth was higher for LDA + UFH + IVIg as compared to LDA or LDA + heparin, and for LDA + corticosteroids as compared to LDA or LDA + LMWH. No treatment was associated with an increased risk of bleeding, thrombocytopenia or osteopenia.

**Supplementary Information:**

The online version contains supplementary material available at 10.1007/s11739-020-02609-4.

## Introduction

Antiphospholipid syndrome (APS) is defined as the occurrence of thrombotic events (thrombotic APS) and/or of recurrent pregnancy morbidity (obstetric APS), in the presence of antiphospholipid antibodies (aPLs), namely lupus anticoagulant (LA), anticardiolipin antibodies (aCL), or anti-β2 glycoprotein-I (aβ2GPI) antibodies, detected on two or more occasions at least 12 weeks apart [1].

In particular, obstetric APS is defined as the presence of aPL positivity and the occurrence of clearly set pregnancy complications, according to the current international criteria [2]. However, in real life, clinicians often face patients with aPL positivity presenting obstetric complications other than those mentioned in the classification criteria [2–6].

Obstetric APS symptoms might be heterogeneous and could involve almost any medical specialty, but the complex management of APS traditionally requires the intervention of internists, in close collaboration with rheumatologists, immunologists, obstetricians and gynaecologists.

In the past, patients with obstetric APS were sometimes advised not to conceive because of the high rate of adverse pregnancy outcomes, including early or late pregnancy losses, placenta-mediated complications, preterm birth, and fetal growth restriction (FGR) [4, 7]. Conversely, in the last 20 years the growing knowledge in the pathogenesis of APS-mediated pregnancy complications and the improvement in the overall management of the disease have paved the way for obstetric APS women to consider the possibility of having children [8].

Patients with obstetric APS usually should plan pregnancy in accordance with clinicians and obstetrician–gynecologists, in order to set up the most appropriate pharmacological strategy in the period before, during and after pregnancy.

Particularly, in the gestational period specific prophylactic treatments should be considered to prevent aPL-mediated placental insufficiency and related complications [9].

Indeed, aPL seem to cause placenta insufficiency by promoting trophoblast apoptosis, by affecting syncytialization, and by downregulating trophoblast invasion. Besides, aPL can also trigger the inflammation of trophoblastic tissues, promoting a pro-inflammatory state in the vascular wall, which in turn leads to a pro-thrombotic state and to placenta insufficiency [10–12].

Among placenta-mediated complications, FGR is defined as an impairment of fetal growth, usually based on the discrepancy between actual and expected fetal ultrasound biometric measurements for a given gestational age (Table S1) [13–16]. Foetuses with FGR do not achieve the expected, genetically predetermined growth potential, mostly as a result of placental dysfunction. In this pathological condition, placenta fails to deliver an adequate supply of oxygen and nutrients to the developing fetus, due to an impaired utero-placental circulation [17]. As earlier and more severe is FGR, as higher is the risk of an impaired intrauterine fetal wellbeing, with short- and long-term consequences, including an increased risk of stillbirth and postnatal mortality [18, 19]. Moreover, also when newborns affected by FGR survive, they still present a high risk of complications in childhood, adolescence and adulthood [20], in terms of impaired neurodevelopment [21, 22] and cardiovascular and metabolic complications [23].

In the last years, many data have been accumulating on the benefits and harms of various pharmacological interventions in pregnant women with obstetric APS. To date, the treatment strategies to prevent APS-related obstetric complications are mainly based on vasoactive treatments such as antiplatelet and/or anticoagulant therapies [namely low-dose aspirin (LDA), and/or unfractionated heparin (UFH) or low molecular weight heparin (LMWH)] [24], and on agents with immunomodulatory effects, such as hydroxychloroquine (HCQ) and corticosteroids [25, 26].

According to the current European League Against Rheumatism (EULAR) recommendations [24], in pregnant women with criteria or non-criteria obstetric APS, treatment with LDA or LDA plus heparin at prophylactic dosage is recommended.

These treatments have a long history of use in obstetric APS, as supportive therapy to counteract the prothrombotic effect of aPL. Traditionally, internists are largely confident with these medications, mainly due to the long-time experience with their use and to the availability of a wide literature supporting their efficacy and safety.

Nevertheless, around 20% of women do not benefit of these treatments [27, 28], particularly in case of triple antibody positivity [29]. In women with criteria obstetric APS with recurrent pregnancy complications despite use of LDA + heparin at prophylactic dosage, increasing heparin dose to therapeutic dosage or addition of HCQ or low-dose corticosteroids can be considered. In pregnant APS women poorly controlled by these therapies, growing literature evidence suggests promising alternative pharmacological approaches, including the use of intravenous immunoglobulins (IVIg) [30] or plasmapheresis [31].

Although the association of LDA and heparin is known to increase live birth rate in obstetric APS women [32], no conclusive evidence exists on the relative benefits and risks of pharmacological interventions for the prevention of FGR in pregnant women with APS, and the use of LDA, heparin or their combination is still debated among clinicians. Indeed, no clinical study specifically focused on FGR as the primary study endpoint and sample size is therefore often underpowered for this outcome.

On this basis, this systematic review and network meta-analysis (NetMA) aimed to summarise literature data on the efficacy and safety of different pharmacological treatments for the prevention of FGR in pregnant women with criteria or non-criteria obstetric APS, with the final aim of providing clinicians updated and clear evidence to guide the interdisciplinary management of pregnancy patients with obstetric APS.

## Methods

### Search strategy and selecting criteria

We conducted a systematic review and NetMA by electronically searching PubMed and Embase databases for studies published from inception until July 1, 2020. The search strategies for PubMed and Embase are reported in Table S2. Additional related studies were sought by reviewing the reference lists of relevant articles.

We included only randomized controlled trials (RCTs) and prospective observational cohort studies, published in English as full-text articles. We selected studies performed on singleton gestating women affected by obstetric APS, with or without thrombotic APS, who met the international criteria for APS diagnosis [2, 33]. We also included studies on pregnant women with non-criteria obstetric APS, defined as patients who were APL‑positive but presented non-criteria clinical manifestations, such as two consecutive unexplained miscarriages at <10 WOG or three or more miscarriages of non-sequential pregnancies. Studies including cases of fetal genetic or chromosomal anomalies, fetal malformations, multiple pregnancies, congenital intrauterine infections, maternal history of drug or alcohol abuse, maternal uterine malformations, or presence of disorders other than APS and/or concomitant SLE requiring the use of antithrombotic agents outside pregnancy, were excluded.

Regarding interventions, we included studies on women treated with UFH or LMWH at prophylactic dosage, LDA, HCQ, corticosteroids or IVIg, either as monotherapy or in association, compared to each other or versus placebo or no treatment (defined as control).

The primary outcome of this NetMA was FGR, and only studies evaluating the effect of pharmacological interventions on this outcome were included.

We also considered the following secondary efficacy outcomes: (i) fetal or neonatal death, defined as pregnancy loss or neonatal death, at any time during pregnancy or in the perinatal period; and (ii) preterm birth, defined as a birth at <37 WOG.

All other maternal, fetal and neonatal outcomes reported in the included studies were narratively summarized.

Three investigators (MLU, AB and IM) independently selected the studies and extracted data related to demographic information, diagnosis, laboratory parameters, obstetric history, index pregnancy, pharmacological interventions, pregnancy outcomes, and maternal and neonatal complications. Additional data related to the study design, year of publication and country in which participants were recruited were recorded, as well.

### Quality assessment of the included studies

The risk of bias for the eligible studies was assessed by two independent reviewers (MLU and AB) using the Cochrane Collaboration risk of bias tool available in RevMan 5.3, and the Newcastle–Ottawa Quality Assessment scale for randomized trials and observational cohort studies, respectively. To assess the risk of reporting bias, registered details of selected clinical trials were sought in the Clinicaltrials.gov database, or in other registry platforms reported by the authors of the studies.

### Data synthesis and statistical analysis

For studies published more than once (i.e., duplicates) only the most informative and complete report was included. All outcomes considered were dichotomous. For each outcome, we considered the number of reported events over the number of patients receiving at least one dose of the study intervention (either treatment or no-treatment). For each efficacy outcome, a NetMA was performed within a frequentist framework to estimate the Odds Ratios (ORs) and 95% confidence intervals (95% CIs). The Mantel–Haenszel method was used for the fixed effect models, if tests of heterogeneity were not significant. If statistical heterogeneity was observed, random effects models were used. Analyses were conducted using the “network” and “network graphs” packages in Stata (StataCorp, version 14.0). The *I*2 statistic was used to assess the heterogeneity of pairwise meta-analyses. To evaluate the presence of inconsistency locally in the NetMA, we used the node-splitting approach [34]. To check the assumption of consistency in the entire network, we used the ‘design-by-treatment’ model using the ‘network’ command in STATA [35], which accounts for different sources of inconsistency.

### Registration

This study is a subgroup analysis of a study registered in PROSPERO, number CRD42019122831.

## Results

### Literature search outcomes

**Fig. 1:**
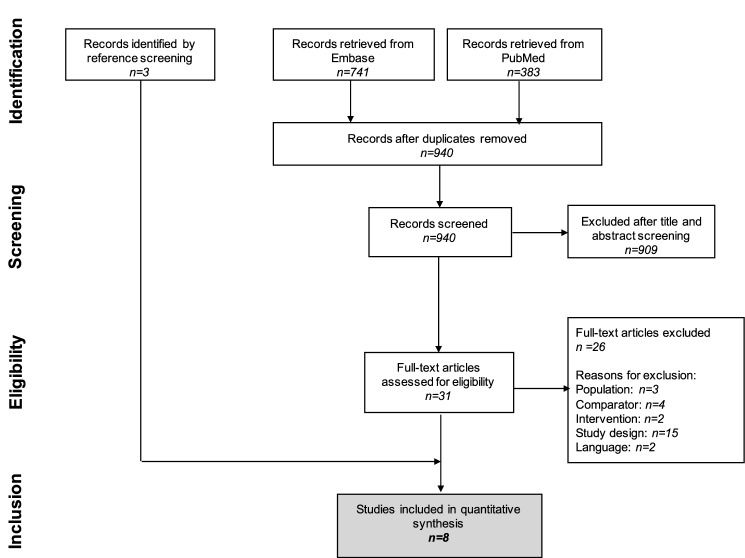
Flow chart of the systematic literature review

We identified 1124 references through electronic searches of PubMed (*n* = 383) and Embase (*n* = 741). After removing 184 duplicates, 940 references were screened. We excluded 909 irrelevant references by reading titles and abstracts. Thirty-one references were retrieved for further assessment. Of them, 26 were excluded for the reasons listed in Fig. 1, whereas five were identified as includible. Other three includible studies were identified by screening the references of the 31 studies read as full-text articles. Overall, eight studies met the inclusion criteria (5 RCTs and 3 prospective cohort studies), for a total of 395 pregnant patients [36–43]. The reference flow is summarised in Fig. 1.

### Study characteristics

The characteristics of the included studies are summarised in Table 1 and more details are provided in Table S3. Six studies included women with diagnosis of obstetric APS with or without thrombotic manifestations, diagnosed according to the international criteria for APS [36–38, 40–42], whereas two studies included women with aPL positivity and non-criteria obstetric complications [39, 43]. In one study [39], two women with APS and diagnosis of SLE were included.

**Table 1:** Characteristics of the included studies

First author, year	Study design; country; study size	Inclusion criteria	Compared interventions	Evaluated outcomes	Results (as reported in the studies)
Mohamed, 2014	Prospective non-randomized trial; Egypt; *n* = 70	Obstetric (± thrombotic) APS	LDA + LMWH: *n* = 47 LDA: *n* = 23	Efficacy outcomes: (a) IUGR (< 10th percentile) (b) Miscarriage (c) Preterm birth < 37 WOG Adverse events: (d) Thrombocytopenia	Efficacy outcomes: (a) 5/43 vs 5/15 among live births (b) 4/47 vs 8/23 (c) 6/43 vs 3/15 among live births Adverse events: (d) 0/47 vs 0/23
Fouda, 2011	RCT; Egypt; *n* = 60	Obstetric APS	LDA + LMWH: *n* = 30 LDA + UFH: *n* = 30	Efficacy outcomes: (a) IUGR (< 10th percentile) (b) First trimester miscarriage (c) Second trimester miscarriage (d) IUFD (e) Preterm labor Adverse events: (f) Osteoporotic fractures (g) Excessive bleeding (h) Thrombocytopenia (i) Subcutaneous bruises (j) Skin allergy (k) Neonatal bleeding	Efficacy outcomes: (a) 1/24 vs 2/20 (b) 6/30 vs 9/30 (c) 0/30 vs 1/30 (d) 0/24 vs 0/20 (e) 3/24 vs 2/20 Adverse events: (f) 0/30 vs 0/30 (g) 0/30 vs 0/30 (h) 0/30 vs 0/30 (i) 3/30 vs 3/30 (j) 0/30 vs 1/30 (k) 0/24 vs 0/20
Noble, 2005^a^	Prospective trial; USA; *n* = 46	Obstetric APS	LDA + LMWH: *n* = 23 LDA + UFH: *n* = 23	Efficacy outcomes: (a) IUGR (< 10th percentile) (b) Pregnancy loss (any time) (c) Preterm birth Adverse events: (d) Minor bleeding (e) Major bleeding at birth (f) Bone fractures (g) Thrombocytopenia	Efficacy outcomes: (a) 1/21 vs 1/20 among live births (b) 2/23 vs 3/23 (c) 2/21 vs 2/20 Adverse events: (d) Not extractable (e) 0/23 vs 0/23 (f) 0/23 vs 0/23 (g) 0/23 vs 0/23
Branch, 2000	RCT; USA; *n* = 16	Obstetric and/or thrombotic APS or high-risk aPL carriers	LDA + UFH + IVIg: *n* = 7 LDA + UFH + placebo: *n* = 9	Efficacy outcomes: (a) IUGR (≤ 10th percentile) (b) Pregnancy loss (any time) (c) Preterm birth (< 37 WOG) Adverse events: (d) Thrombocytopenia (e) Bleeding (f) Osteopenic fractures	Efficacy outcomes: (a) 1/7 vs 3/9 (b) 0/7 vs 0/9 (c) 7/7 vs 3/9 Adverse events: (d) 1/7 vs 0/9 (e) 0/7 vs 0/9 (f) 0/7 vs 0/9
Rai, 1997	RCT; UK; *n* = 90	Primary obstetric APS	LDA: *n* = 45 LDA + UFH: *n* = 45	Efficacy outcomes: (a) IUGR (< 10th percentile) (b) Miscarriages (c) Preterm birth (< 37 WOG) Adverse events: (d) Thrombocytopenia (e) Vertebral fracture	Efficacy outcomes: (a) 1 vs 3 (b) 26/45 vs 13/45 (c) 4/19 vs 8/32 among live births Adverse events: (d) 0/45 vs 0/45 (e) 0/45 vs 0/45
Kutteh, 1996	RCT; USA; *n* = 50	Primary obstetric APS	LDA + UFH: *n* = 25 LDA: *n* = 25	Efficacy outcomes: (a) IUGR (< 10th percentile) (b) Pregnancy loss (c) Preterm birth Adverse events: (d) Minor bleeding (e) Preeclampsia (f) Major bleeding (g) Fractures	Efficacy outcomes: (a) 3/20 vs 1/11 for live births (b) 5/25 vs 14/25 (c) 3/20 vs 1/11 among live births Adverse events: (d) 3/20 vs 1/11 (e) 2/20 vs 1/11 (f) 0/20 vs 0/11 (g) 0/20 vs 0/11
Silver, 1993	RCT; USA; *n* = 34	Obstetric ± thrombotic APS	LDA: *n* = 22 LDA + prednisone (20 mg/day, range 10–40 mg/day): *n* = 12	Efficacy outcomes: (a) SGA (< 10th percentile) (b) Pregnancy loss (any time) (c) Preterm birth (< 37 wog) Adverse events: *Not reported*	Efficacy outcomes: (a) 0/22 vs 0/12 (b) 0/22 vs 0/12 (c) 3/22 vs 8/12
Hasegawa, 1992	Prospective observational study; Japan; *n* = 29	aPL positivity + history of 2 + recurrent pregnancy losses	LDA + Prednisolone (40 mg/day for 4 weeks and tapering): *n* = 17 Untreated: *n* = 12	Efficacy outcomes: (a) FGR (birth weight < -1.5 SD**)** (b) Miscarriage or fetal death (c) Neonatal death (d) Preterm birth Adverse events: *Not reported*	Efficacy outcomes: (a) 4/13 vs 5/6 among not aborted (b) 4/17 vs 9/12 (c) 0/17 vs 2/12 (d) *Not reported*

*aPL* antiphospholipid antibodies; *APS* antiphospholipid syndrome; *FGR* fetal growth restriction; *IUFD* intrauterine fetal death; *IUGR* intrauterine growth retardation; *IVIg* intravenous immunoglobulin; *LDA* low dose aspirin; *LMWH* low molecular weight heparin; *RCT* randomized controlled trial; *SGA* small for gestational age; *SLE* systemic lupus erythematosus; *UFH* unfractionated heparin; *WOG* weeks of gestation

^a^Total sample size: 50 patients; 4 cases (2 in each treatment group) had abnormal karyotypes and were excluded from the meta-analysis

**Fig. 2:**
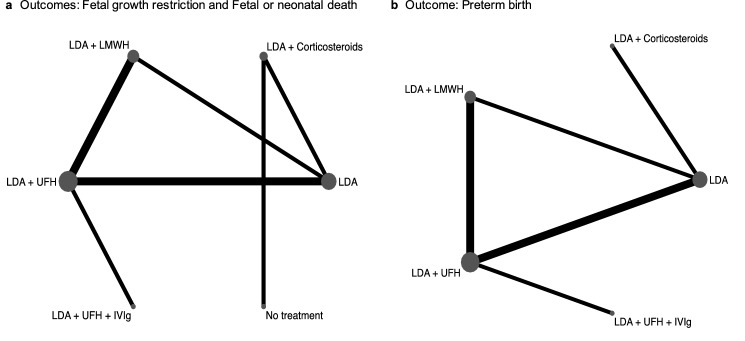
Network map of comparisons for the outcomes fetal growth restriction and fetal or neonatal death (**a**) and for the outcome preterm birth (**b**) IVIg: intravenous immunoglobulin; LDA: low dose aspirin; LMWH: low molecular weight heparin; UFH: unfractionated heparin

Regarding pharmacological interventions, two studies compared LDA + LMWH vs LDA + UFH [37, 38], two LDA + UFH vs LDA [40, 41], one LDA + LMWH vs LDA [36]. One study compared LDA + UFH + placebo vs LDA + UFH + IVIg [39], one compared LDA + prednisolone vs no treatment [43], and the last one compared LDA + prednisone vs LDA [42]. In all studies, LDA was administered orally once daily, at a dosage <100 mg/day. Heparin (LMWH and UFH) was administered at prophylactic dosage, according to routine clinical practice. No study on HCQ was found. The specific comparisons between treatments in the different studies are illustrated in Fig. 2a.

Overall, 132 patients (34%) were treated with LDA + UFH, 115 (29%) with LDA, 100 (25%) with LDA + LMWH, 29 (7%) with LDA + corticosteroids, 7 (2%) with LDA + UFH + IVIg, and 12 (3%) were untreated.

### Risk of bias assessment

The assessment of the risk of bias is reported in the Table S4 and Figure S1. Considering non-randomized studies (Table S4), one study was judged at low risk of bias for all quality domains [36] and two studies were judged at high risk of selection bias and at low risk of bias for all other quality items [38, 43]. As for randomized studies (Figure S1), three studies were judged at high risk of selection bias due to random sequence generation and/or to allocation concealment [39–41]. Regarding performance and allocation bias, most studies (4/5) were judged at unclear risk, as they were open-label studies [37, 40–42]. However, blinding of participants or personnel was considered to be difficult to perform, due to the different route of administration of the study interventions, and was considered unlikely to have influenced the study outcomes. One study was considered at high risk of attrition bias [42], and most studies (4/5) were considered at high risk of reporting bias, as no published protocol was available [39–42].

### Outcomes

FGR was the primary outcome of this NetMA, evaluated in all studies included. Secondary efficacy outcomes evaluated in the quantitative synthesis included fetal or neonatal death and preterm birth. All additional pregnancy and maternal outcomes are reported in Table S3.

Overall, 36 cases of FGR were reported among the 395 treated pregnancies [9.1% (95% CI 6.6–12.4)]. The specific comparisons between treatments evaluated in the different studies are illustrated in Fig. 2a. Both mixed and direct evidence showed no statistically significant difference in terms of FGR risk among treatments, nor between active treatments and no treatment (Table 2; Figure S2).

**Table 2:** Comparisons derived from direct and mixed evidence for the efficacy outcomes fetal growth restriction, fetal or neonatal death, and preterm birth

*Outcome 1—Fetal growth restriction*					
LDA (4 studies; 115 patients)	0.49 (0.13–1.86)	3.00 (0.58–15.42)	–	–	–
0.62 (0.19–2.04)	LDA + LMWH (3 studies; 100 patients)	1.50 (0.24–9.33)	–	–	–
1.87 (0.49–7.11)	3.02 (0.75–12.24)	LDA + UFH (5 studies; 132 patients)	–	0.43 (0.04–5.06)	–
1.80 (0.03–96.12)	2.90 (0.05–194.65)	0.96 (0.01–63.87)	–	–	1.77 (0.39–8.00)
0.62 (0.04–304.49)	1.01 (0.06–18.14)	0.33 (0.03–4.19)	0.35 (0.00–46.60)	LDA + UFH + IVIg (1 study; 7 patients)	–
4.17 (0.06–304.49)	6.74 (0.08–577.86)	2.23 (0.02–199.29)	2.23 (0.47–11.54)	6.69 (0.04–1160.82)	No treatment (1 study; 12 patients)
*Outcome 2—Fetal or neonatal death*					
LDA (4 studies; 115 patients)	0.25 (0.07–0.90)	0.45 (0.24–0.86)	NC	–	-
0.15 (0.06–0.38)	LDA + LMWH (3 studies; 100 patients)	1.62 (0.62–4.27)	–	–	-
0.27 (0.14–0.52)	1.75 (0.76–4.02)	LDA + UFH (5 studies; 132 patients)	–	NC	-
1.80 (0.03–95.99)	11.66 (0.20–689.79)	6.68 (0.12–376.77)	LDA + corticosteroids (2 studies; 29 patients)	–	3.90 (1.00–15.21)
0.34 (0.01–20.33)	2.21 (0.04–136.21)	1.27 (0.02–71.63)	0.19 (0.00–56.96)	LDA + UFH + IVIg (1 study; 7 patients)	-
64.22 (0.64–6466.54)	416.91 (3.79–45,836.27)	238.73 (2.26–25,194.27)	35.74 (3.46–368.77)	188.44 (0.40–89,523.29)	No treatment (1 study; 12 patients)
*Outcome 3—Preterm birth*					
LDA (4 studies; 115 patients)	0.98 (0.22–4.27)	2.21 (0.73–6.71)	4.89 (1.09–21.95)	–	
1.57 (0.50–4.90)	LDA + LMWH (3 studies; 100 patients)	0.80 (0.20–3.14)	–	–	
1.85 (0.69–4.94)	1.18 (0.39–3.58)	LDA + UFH (5 studies; 132 patients)	–	3.00 (0.56–16.01)	
12.67 (2.29–70.02)	8.09 (1.04–63.14)	6.85 (0.95–49.17)	LDA + corticosteroids (1 study; 12 patients)	–	
51.54 (1.91–1388.37)	32.91 (1.17–922.69)	27.85 (1.20–645.95)	4.07 (0.10–166.38)	LDA + UFH + IVIg (1 study; 7 patients)	

*IVIg* intravenous immunoglobulin; *LDA* low dose aspirin; *LMWH* low molecular weight heparin; *NC* not calculable; *UFH* unfractionated heparin

The secondary outcome fetal or neonatal death was evaluated in all the included studies (Fig. 2a). Overall, 106 cases of fetal or neonatal death were reported among the 395 treated pregnancies [26.8% (95% CI 22.7–31.4)]. Both mixed and direct evidence showed that treatment with LDA + LMWH or LDA + UFH were associated with a significant lower risk of fetal or neonatal death as compared to LDA alone (OR (95%CI) from mixed evidence of 0.15 (0.06–0.38) and of 0.27 (0.14–0.52) for LDA + LMWH vs LDA and for LDA + UFH vs LDA, respectively) (Table 2). Furthermore, the risk of fetal or neonatal death resulted significantly higher in untreated patients as compared to patients treated with LDA + corticosteroids [OR from mixed comparison: 35.74 (3.46–368.77)].

The secondary outcome preterm birth was evaluated in 7/8 included studies (Fig. 2b) [36–42]. Overall, 55 cases of preterm birth occurred among the 366 women for whom this outcome was reported [15.0% (95% CI 11.7–19.1)]. Mixed evidence showed that the association of LDA + UFH + IVIg was associated with a significantly higher risk of preterm birth as compared to treatment with LDA alone, LDA + LMWH, or LDA + UFH (OR of 51.54 (1.91–1388.37), 32.91 (1.17–922.69) and 27.85 (1.20–645.95), respectively) (Table 2). Similarly, treatment with LDA + corticosteroids resulted to be associated with a significantly higher risk of preterm birth as compared to both LDA monotherapy (OR from mixed evidence 12.67 (2.29–70.02)) and LDA + LMWH (8.09 (1.04–63.14)).

No evidence of statistically significant overall or loop-specific inconsistency, or heterogeneity in pairwise meta-analyses, was found for the three outcomes.

Regarding safety outcomes, only one case of thrombocytopenia was reported, in a woman treated with LDA + UFH + IVIg. Four cases of minor bleeding (including haematuria, nose or gum bleeding, and bleeding at injection site) were reported, of whom three in patients on LDA + UFH and one in a patient with LDA. No cases of major bleeding or osteoporotic fractures were reported.

## Discussion

To our knowledge, this systematic review and NetMA represents the first synthesis of evidence on prophylactic treatments for the prevention of FGR in patients with criteria and non-criteria obstetric APS.

Adequate periconception management is still an unmet need for many patients with obstetric APS. Despite many data supporting the use of various therapeutic options for the prevention of APS-related obstetric complications, no consensus exists on the role of pharmacological interventions for the prevention of FGR in pregnant women with APS, and the use of LDA, heparin or their combination is still debated among clinicians. Indeed, clear evidence supporting the use of one treatment over another is hampered by the limited number of trials of small sample size and by many (often discordant) data from heterogeneous clinical experiences.

In this context, this NetMA was aimed at providing a unique synthesis of literature evidence on prophylactic treatments for the prevention of FGR in patients with criteria or non-criteria obstetric APS.

Our study included eight studies on 395 pregnant women with criteria and non-criteria obstetric APS, treated with five different pharmacological approaches (LDA, LDA + LMWH, LDA + UFH, LDA + corticosteroids, LDA + UFH + IVIg) or with placebo or no treatment, for the prevention of adverse pregnancy outcomes.

Overall, we found no statistically significant difference in the risk of FGR among active therapies, nor between active therapies and no treatment.

Conversely, we found that the association of LDA and heparin (either LMWH or UFH) was significantly more effective than LDA alone in preventing fetal or neonatal death. Similarly, women treated with LDA + corticosteroids resulted at significantly lower risk of FGR compared to untreated women. However, estimates were largely imprecise, particularly for LDA + corticosteroids, and most studies were judged at high or unclear risk of bias for all quality domains. Thus, the confidence in the evidence regarding this intervention should be considered low.

Regarding risk of preterm birth, results from this NetMA suggest that treatment with LDA + UFH + IVIg is associated with a higher risk as compared to treatment with LDA alone or LDA plus heparin (either LMWH or UFH). However, also in this case estimates were largely imprecise for all three comparisons and derived only from indirect evidence.

Although underreporting of adverse events cannot be excluded, especially given the nature of the studies included (RCT and prospective observational), our results indicate no evident risk of thrombocytopenic, major bleeding or osteoporotic complications in women treated with these interventions.

Our results regarding the higher efficacy of LDA plus heparin as compared to LDA alone in preventing pregnancy mortality are in line with current literature [32]. Indeed, the last EULAR recommendations for the management of obstetric APS recommend the use of LDA plus heparin, rather than of LDA alone, in pregnant women with criteria or non-criteria obstetric APS [24]. In women with recurrent pregnancy complications despite the use of LDA plus heparin, current EULAR recommendations suggest the addition of HCQ or low-dose corticosteroids [24]. Unexpectedly, none of the included study evaluated HCQ as a therapeutic strategy for the prevention of recurrent pregnancy complications related to APS, despite the current recommendations list HCQ as a valuable option for APS management.

Furthermore, despite the absence of RCTs, growing evidence derived from observational studies suggest a beneficial role of IVIg, in addition to standard prophylaxis, in preventing obstetric morbidity related to APS [30]. By contrast, the results from our NetMA did not identify a beneficial role of IVIg, in association with LDA and heparin, in preventing APS-related obstetric morbidity and mortality. However, these results are likely to be influenced by the high heterogeneity in terms of clinical and obstetric history among women in the different studies and the small number (*n* = 7) of patents treated with this intervention.

Our NetMA has some limitations that deserve discussion. First, none of the included studies considered FGR as the primary outcome, and sample size of each study was therefore calculated on other outcomes rather than on FGR. This might have accounted, at least in part, for the largely imprecise evidence coming from both direct and indirect comparisons in this NetMA. Second, the pharmacological interventions significantly varied among studies; as a consequence, evidence for the specific comparisons between two treatments only came from few studies, thus failing to provide solid results. Third, the wide temporal window in which studies were conducted (1992–2014) might have affected the transitivity assumption on which the NetMA approach is based; indeed, general management of APS as well as the criteria for APS diagnosis significantly varied over the last decades.

Another shortcoming of this NetMA is that all studies were performed in obstetric/gynecologic centers, rather than in rheumatologic or immunologic units highly qualified for the management of APS.

Nevertheless, this NetMA represents the unique synthesis of literature evidence specifically focusing on prophylactic treatments for the prevention of FGR in patients with criteria or non-criteria obstetric APS.

This NetMA included more than 390 pregnant women with obstetric APS, either criteria or non-criteria, while aPL carriers were excluded. This choice guaranteed a homogeneity in terms of the study population, as all women presented a past history of pregnancy morbidity and/or mortality. Moreover, all studies included in this NetMA were prospective (either randomized or observational), thus assuring that relevant information regarding the therapeutic approach and the pregnancy outcomes was prospectively recorded.

Taken together, the results from this NetMA failed to provide conclusive evidence on the most effective therapeutic option(s) for FGR prevention in criteria and non-criteria obstetric APS patients.

Based on our findings, the association of LDA and heparin (either LMWH or UFH) should be preferred to LDA alone for preventing APS-related obstetric mortality, but does not provide additional benefits in terms of FGR prevention.

These results rise the lack of clear high-quality evidence to guide clinicians in the management of the various pregnancy complications that might occur in obstetric APS patients and that, outside fetal and perinatal mortality, might account for a considerable burden of neonatal morbidity.

In this context, new high-quality and pragmatic trials are advocated to specifically compare the benefits of different active pharmacological therapies for FGR prevention, with a specific focus on the role of immunomodulating/immunosuppressants agents, as well as epidemiological safety studies to address the actual safety of the treatments in a real-world setting. Results from these trials might help future clinical decision-making, particularly for the prevention of FGR recurrence in obstetric APS patients with history of placenta-mediated pregnancy morbidity.

## Supplementary Information

Below is the link to the electronic supplementary material.

Supplementary file1 (DOCX 171 KB)

## Data Availability

The full dataset on which meta-analyses were run is available upon written request to the corresponding author. The STATA code for this NetMA is available upon written request to the corresponding author.
