# Supplementary material for: Comparison of treatments for the prevention of fetal growth restriction in obstetric antiphospholipid syndrome: a systematic review and network meta-analysis
Source: Intern Emerg Med. 2021 Jan 21;16(5):1357–67. doi: 10.1007/s11739-020-02609-4 (PMC8310508; doi:10.1007/s11739-020-02609-4)
Supplement: Supplementary file 1 — Supplementary file1 (DOCX 171 KB) [file 11739_2020_2609_MOESM1_ESM.docx]

|  | **Definition** | **Notes** |
| --- | --- | --- |
|  |  |  |
| Fetal growth restriction (FGR)/ Intrauterine growth retardation (IUGR) | - “Fetuses with an estimated fetal weight that is less than the 10^th^ percentile for gestational age” - (American College of Obstetricians and Gynecologists (ACOG) 2019 [1])   Several definitions have been used for IUGR, including but not limited to estimated fetal weight <25, <15, <10, <5, <3, <2.5, and <1 percentile for gestational age, estimated weight less than 2 standard deviations below the mean weight, abdominal circumference <10 % for gestational age [2] | The definition does not take into account the individualized growth potential of each fetus.  This might result in the fail to  identify larger fetuses that have not achieved their growth potential and may be at risk of adverse outcomes, or in the misdiagnosis of constitutionally small fetuses [1] |
| Small for gestational age (SGA) | “Newborns whose birth weight is less than the 10th percentile for gestational age” (American College of Obstetricians and Gynecologists (ACOG) 2019 [1]) | The definition of SGA will include a proportion of babies (18-22%) who are constitutionally small but healthy [3,4] |

**Table S1** Definition of fetal growth restriction, intrauterine growth retardation, and small for gestational age

1. ACOG Practice Bulletin No. 204: Fetal Growth Restriction. *Obstet. Gynecol.* **2019**, *133*, e97–e109.

2. Suhag, A.; Berghella, V. Intrauterine Growth Restriction (IUGR): Etiology and Diagnosis. *Curr. Obstet. Gynecol. Rep.* **2013**, *2*, 102–111.

3. McCowan, L.M.; Figueras, F.; Anderson, N.H. Evidence-based national guidelines for the management of suspected fetal growth restriction: comparison, consensus, and controversy. *Am. J. Obstet. Gynecol.* 2018, *218*, S855–S868.

4. McCowan, L.M.E.; Harding, J.E.; Stewarf, A.W. Customised birthweight centiles predict SGA pregnancies with perinatal morbidity. *BJOG An Int. J. Obstet. Gynaecol.* **2005**, *112*, 1026–1033.

**Table S2** Search strategies for PubMed and Embase

| Search strategy for PubMed | ((fetal growth restriction) OR (intrauterine growth restriction) OR (small for gestational age)) AND (antiphospholipid) |
| --- | --- |
| Search strategy for Embase | ('fetal growth restriction'/exp OR 'fetal growth restriction' OR (fetal AND ('growth'/exp OR growth) AND restriction) OR 'intrauterine growth restriction'/exp OR 'intrauterine growth restriction' OR (intrauterine AND ('growth'/exp OR growth) AND restriction) OR 'small for gestational age'/exp OR 'small for gestational age' OR (small AND for AND gestational AND ('age'/exp OR age))) AND antiphospholipid NOT ([editorial]/lim OR [review]/lim) |

**Table S3** Detailed characteristics and outcomes of the included studies

| **First author, year** | **Study design; country; study size** | **Inclusion criteria** | **Compared interventions** | **Evaluated outcomes** | **Results (as reported in the studies)** |
| --- | --- | --- | --- | --- | --- |
| Mohamed, 2014 | Prospective non-randomized trial; Egypt; n=70 | Obstetric (± thrombotic) APS | LDA + LMWH: n=47  LDA: n=23 | Fetal outcomes:   1. **IUGR (<10th percentile)** 2. Miscarriage 3. Live births 4. Mean WOG at birth 5. Birth weight (g) 6. Preterm birth <37 WOG   Maternal outcomes:   1. Preeclampsia 2. Thromboembolism   Neonatal outcomes:   1. Admission to NICU 2. Congenital abnormalities   Adverse events:   1. Thrombocytopenia | Fetal outcomes:   1. **5/43 vs 5/15 among live births** 2. 4/47 vs 8/23 3. 43/47 vs 15/23 4. 37.9 ± 1.8 vs 36.1 ± 2.4 5. 3252 ± 459 vs 2907 ± 618 6. 6/43 vs 3/15 among live births   Maternal outcomes:   1. 3/43 vs 6/15 with live births 2. 0/47 vs 0/23   Neonatal outcomes:   1. 6/43 vs 6/15 among live births 2. 0/43 vs 0/15 among live births   Adverse events:   1. 0/47 vs 0/23 |
| Fouda, 2011 | RCT; Egypt; n=60. | Obstetric APS | LDA + LMWH: n=30  LDA + UFH: n=30 | Fetal outcomes:   1. **IUGR (<10th percentile)** 2. First trimester miscarriage 3. Second trimester miscarriage 4. Live births 5. Preterm labor 6. IUFD 7. Gestational age at birth 8. Birth weight (g)   Maternal outcomes:   1. Preeclampsia   Neonatal outcomes:   1. Congenital abnormalities 2. Admission to NICU   Adverse events:   1. Osteoporotic fractures 2. Excessive bleeding 3. Thrombocytopenia 4. Subcutaneous bruises 5. Skin allergy 6. Neonatal bleeding | Fetal outcomes:   1. **1/24 vs 2/20** 2. 6/30 vs 9/30 3. 0/30 vs 1/30 4. 24/30 vs 20/30 5. 3/24 vs 2/20 6. 0/24 vs 0/20 7. 38.54 ± 1.41 vs 38.15 ±1.84 8. 3183 ± 382 vs 3087 ± 563   Maternal outcomes:   1. 2/24 vs 1/20   Neonatal outcomes:   1. 0/24 vs 0/20 2. 2/24 vs 2/20   Adverse events:   1. 0/30 vs 0/30 2. 0/30 vs 0/30 3. 0/30 vs 0/30 4. 3/30 vs 3/30 5. 0/30 vs 1/30 6. 0/24 vs 0/20 |
| Noble, 2005∞ | Prospective trial; USA; n=46 | Obstetric APS | LDA + LMWH: n=23  LDA + UFH: n=23 | Fetal outcomes:   1. **IUGR (<10^th^ percentile**) 2. Live births 3. Preterm birth   Maternal outcomes:   1. Preeclampsia 2. DVT   Adverse events:   1. Minor bleeding 2. Major bleeding at birth 3. Bone fractures 4. Thrombocytopenia | Fetal outcomes:   1. **1/21 vs 1/20 among live births** 2. 21/23 vs 20/23 3. 2/21 vs 2/20   Maternal outcomes:   1. 0/23 vs 0/23 2. 0/23 vs 0/23   Adverse events:   1. Not extractable 2. 0/23 vs 0/23 3. 0/23 vs 0/23 4. 0/23 vs 0/23 |
| Branch, 2000 | RCT; USA; n=16 | Obstetric and/or thrombotic APS or high-risk aPL carriers | LDA + UFH + IVIg: n=7  LDA + UFH + Placebo: n=9 | Fetal outcomes:   1. **IUGR (≤10th percentile)** 2. Live births 3. Preterm birth (<37 WOG) 4. Oligohydramnios 5. Fetal distress   Maternal outcomes:   1. Preeclampsia 2. Eclampsia   Neonatal outcomes:   1. Admission to NICU 2. Respiratory distress syndrome   Adverse events:   1. Thrombocytopenia 2. DVT (4 weeks after birth) 3. Bleeding 4. Osteopenic fractures | Fetal outcomes:   1. **1/7 vs 3/9** 2. 7/7 vs 9/9 3. 7/7 vs 3/9 4. 2/7 vs 2/9 5. 0/7 vs 3/9   Maternal outcomes:   1. 3/7 vs 1/9 2. 0/7 vs 0/9   Neonatal outcomes:   1. 1/7 vs 4/9 2. 0/7 vs 1/9   Adverse events:   1. 1/7 vs 0/9 2. 1/7 vs 0/9 3. 0/7 vs 0/9 4. 0/7 vs 0/9 |
| Rai, 1997 | RCT; UK; n=90 | Primary obstetric APS | LDA: n=45  LDA + UFH: n=45 | Fetal outcomes:   1. **IUGR (<10^th^ percentile)** 2. Miscarriages 3. Live births 4. Preterm birth (<37 WOG) 5. Median birth weight (g)   Maternal outcomes:   1. Pre-eclampsia 2. Thrombotic complications   Neonatal outcomes:   1. Congenital abnormalities 2. Parietal lobe infarction in the newborn   Adverse events:   1. Thrombocytopenia 2. Vertebral fracture | Fetal outcomes:   1. **1 vs 3** 2. 26/45 vs 13/45 3. 19/45 vs 32/35 4. 4/19 vs 8/32 among live births 5. 3080 (range 1300-4350) vs 3330 (1510-4140)   Maternal outcomes:   1. 1/45 vs 0/45 2. 0/45 vs 0/45   Neonatal outcomes:   1. 0/19 vs 0/32 2. 1/19 vs 0/32   Adverse events:   1. 0/45 vs 0/45 2. 0/45 vs 0/45 |
| Kutteh, 1996 | RCT; USA; n=50 | Primary obstetric APS | LDA + UFH: n=25  LDA: n=25 | Fetal outcomes:   1. **IUGR (<10th percentile)** 2. Live births 3. WOG at birth 4. Birth weight (g) 5. Preterm birth 6. Pregnancy loss   Maternal outcomes for women with live births:   1. Thrombocytopenia   Adverse events:   1. Minor bleeding 2. Preeclampsia 3. Major bleeding 4. Fractures | Fetal outcomes:   1. **3/20 vs 1/11 for live births** 2. 20/25 vs 11/25 3. 37.2 ±3.4 vs 37.8 ± 2.1 for live births 4. 2922 ±716 vs 3064 ±628 for live births 5. 3/20 vs 1/11 among live births 6. 5/25 vs 14/25   Maternal outcomes for women with live births:   1. 0/20 vs 0/11   Adverse events:   1. 3/20 vs 1/11 2. 2/20 vs 1/11 3. 0/20 vs 0/11 4. 0/20 vs 0/11 |
| Silver, 1993 | RCT; USA; n=34 | Obstetric ± thrombotic APS | LDA: n=22  LDA + prednisone: n=12 | Fetal outcomes:   1. **SGA (<10th percentile**) 2. Live births 3. Preterm birth (<37 wog)   Maternal outcomes:   1. Placental abruption 2. Endometritis 3. Wound dehiscence 4. DVT 5. Puerperal autoimmune pneumonitis   Adverse events:  *Not reported* | Fetal outcomes:   1. **0/22 vs 0/12** 2. 22/22 vs 12/12 3. 3/22 vs 8/12   Maternal outcomes:   1. 0/22 vs 2/12 2. 0/22 vs 3/12 3. 0/22 vs 1/12 4. 0/22 vs 1/12 5. 1/22 vs 0/12 |
| Hasegawa, 1992 | Prospective observational study; Japan; n=29 | aPL positivity + history of 2+ recurrent pregnancy losses | LDA + Prednisolone: n=17  Untreated: n=12 | Fetal outcomes:   1. **FGR (birth weight < -1.5 SD)** 2. Live births 3. Miscarriage or fetal death 4. Neonatal death   Maternal outcomes:  *Not reported*  Adverse events:  *Not reported* | Fetal outcomes:   1. **4/13 vs 5/6 among not aborted** 2. 13/17 vs 1/12 3. 4/17 vs 9/12 4. 0/17 vs 2/12 |

*∞ Total sample size: 50 patients; 4 cases (2 in each treatment group) had abnormal karyotypes and were excluded from the meta-analysis.*

*aPL: antiphospholipid antibodies; APS: antiphospholipid syndrome; DVT: deep venous thrombosis; FGR: fetal growth restriction; IUFD: intrauterine fetal death; IUGR: intrauterine growth retardation; IVIg: intravenous immunoglobulin; LDA: low dose aspirin; LMWH: low molecular weight heparin; NICU: neonatal intensive care unit; RCT: randomized controlled trial; SGA: small for gestational age; SLE: systemic lupus erythematosus; UFH: unfractionated heparin; WOG: weeks of gestation.*

** Mean ± SD (Standard Deviation); § Mean (range)*

**Table S4** Risk of bias table of non-randomized studies, performed according to the Newcastle-Ottawa scale

|  | **Selection (score)** | | | | **Comparability (score)** | **Exposure (score)** | | |
| --- | --- | --- | --- | --- | --- | --- | --- | --- |
|  | ***Representativeness of Exposed Cohort*** | ***Selection of the Non Exposed Cohort*** | ***Ascertain of exposure*** | ***No Outcome of interest at the start of the study*** | ***Control for Important Factor or Additional Factor*** | ***Assessment of Outcome*** | ***Adequate follow up*** | ***Adequacy of follow up cohorts*** |
| **Hasegawa 1992** | 0  aPL positivity + history of 2+ recurrent pregnancy losses | 1  Drawn from the same community | 1  Prospective study | 1  NA | 1  The two groups were comparable in terms of demographic characteristics and pregnancy history. Management plans were not changed at any time during the study period. | 1  The investigators at birth institution assessed the outcomes. | 1  Treatment was started as soon as pregnancy was confirmed. | 1  No patient was lost to follow-up. |
| **Mohamed 2014** | 1  Obstetric (+/- thrombotic) APS | 1  Drawn from the same community | 1  Prospective study | 1  NA | 1  The two groups were comparable in terms of demographic characteristics and pregnancy history. Management plans were not changed at any time during the study period. | 1  The investigators at birth institution assessed the outcomes. | 1  Treatment was started as soon as pregnancy was confirmed. | 1  No patient was lost to follow-up. |
| **Noble**  **2005** | 1  Obstetric APS | 0  Patients treated in two different centers | 1  Prospective study | 1  NA | 1  The two groups were comparable in terms of demographic characteristics and pregnancy history. Management plans were not changed at any time during the study period. | 1  The investigators at birth institution assessed the outcomes. | 1  Treatment was started as soon as pregnancy was confirmed. | 1  No patient was lost to follow-up. |

*1: study awarded for the considered domain; 0: study not awarded for the considered domain.*

*APS: antiphospholipid syndrome; NA: not applicable*

**Fig. S1** Risk of bias graph assessment for randomized studies, performed according to Cochrane Collaboration’s risk of bias tool

*Green circles: low risk of bias for the considered domain; yellow circles: unclear risk of bias; red circles: high risk of bias; NA: not applicable.*

**Fig. S2** Forest plot on the risk of fetal growth restriction among pharmacological interventions, derived from mixed evidence

*IVIg: intravenous immunoglobulin; LDA: low dose aspirin; LMWH: low molecular weight heparin; UFH: unfractionated heparin*
